# Risk factors and management of intraprocedural rupture during coil embolization of unruptured intracranial aneurysms: role of balloon guiding catheter

**DOI:** 10.3389/fneur.2024.1343137

**Published:** 2024-01-17

**Authors:** Ken Aoki, Yuichi Murayama, Yoshihiro Tanaka, Toshihiro Ishibashi, Koreaki Irie, Michiyasu Fuga, Naoki Kato, Issei Kan, Kengo Nishimura, Gota Nagayama

**Affiliations:** ^1^Department of Neurosurgery, The Jikei University School of Medicine, Tokyo, Japan; ^2^Department of Neurosurgery, Katsushika Medical Center, The Jikei University School of Medicine, Tokyo, Japan; ^3^Division of Epidemiology, Graduate School of Public Health, Shizuoka Graduate University of Public Health, Shizuoka, Japan; ^4^Department of Neurosurgery, The Jikei University Kashiwa Hospital, Chiba, Japan

**Keywords:** endovascular treatment, intraprocedural rupture, unruptured intracranial aneurysm, risk factor, balloon guiding catheter

## Abstract

**Introduction:**

Intraprocedural rupture (IPR) is a serious complication of endovascular coil embolization of unruptured intracranial aneurysms (UIAs). Although outcomes after IPR are poor, methods to prevent subsequent neurological deterioration have not yet been investigated. We evaluated the risk factors and management strategies for IPR, particularly the role of balloon guiding catheters (BGCs) in rapid hemostasis.

**Methods:**

We retrospectively reviewed all UIA cases treated with coil embolization at three institutions between 2003 and 2021, focusing on preoperative radiological data, operative details, and outcomes.

**Results:**

In total, 2,172 aneurysms were treated in 2026 patients. Of these, 19 aneurysms in 19 patients (0.8%) ruptured during the procedure. Multivariate analysis revealed that aneurysms with a bleb (OR: 3.03, 95% CI: 1.21 to 7.57, *p* = 0.017), small neck size (OR: 0.56, 95% CI: 0.37 to 0.85, *p* = 0.007), and aneurysms in the posterior communicating artery (PcomA) (OR: 4.92, 95% CI: 1.19 to 20.18, *p* = 0.027) and anterior communicating artery (AcomA) (OR: 12.08, 95% CI: 2.99 to 48.79, *p* < 0.001) compared with the internal carotid artery without PcomA were significantly associated with IPR. The incidence of IPR was similar between the non-BGC and BGC groups (0.9% vs. 0.8%, *p* = 0.822); however, leveraging BGC was significantly associated with lower morbidity and mortality rates after IPR (0% vs. 44%, *p* = 0.033).

**Discussion:**

The incidence of IPR was relatively low. A bleb, small aneurysm neck, and location on PcomA and AcomA are independent risk factors for IPR. The use of BGC may prevent fatal clinical deterioration and achieve better clinical outcomes in patients with IPR.

## 1 Introduction

Endovascular coil embolization is a well-established treatment for unruptured intracranial aneurysms (UIAs) (1, 2). Intraprocedural aneurysmal rupture (IPR) is a rare but serious complication of endovascular coil embolization, resulting in poor clinical outcomes. Although technical improvements and careful assessment of anatomical and device-related pitfalls can reduce the risk of rupture, a residual risk remains for procedure-related rupture, which has been reported to occur in 1%–10% of coil embolization procedures, with subsequent mortality rates of up to 33% (1–18). Prior studies have investigated the incidence of IPR and related risk factors; however, the technical knowledge required to achieve rapid hemostasis and prevent neurological deterioration remains largely unexplored.

Balloon guiding catheters (BGCs) are commonly used in combination with thrombectomy catheters, such as aspiration catheters or stent retrievers, for treating acute ischemic stroke. In the setting of IPR during coil embolization of aneurysms, BGCs can achieve rapid hemostasis and may prevent fatal outcomes. At our institutions (main and two affiliated university hospitals), we have been using BGCs to treat suitable ruptured aneurysms or UIAs since 2010.

In this study, we investigated the incidence, risk factors, and outcomes of IPR at our institutions and analyzed the role of BGCs in the endovascular treatment of UIAs. To the best of our knowledge, this is the first study to address the role of BGCs in the management of IPR of UIAs.

## 2 Materials and methods

### 2.1 Patients and data collection

This study was approved by the Ethics Committee of The Jikei University School of Medicine [approval number 29–228(8844)]. Informed consent was obtained from the patients for all surgical procedures performed in this study.

We retrospectively reviewed all cases of saccular UIAs initially treated with coil embolization at our university hospitals between January 2003 and March 2021. Cases of mycotic, dissecting, fusiform, traumatic, and pseudoaneurysms, aneurysms related to arteriovenous malformations, dural arteriovenous fistulas, and moyamoya disease, and aneurysms treated with parent artery occlusion and flow diverters were excluded. We also excluded cases of ruptured aneurysms because of the difficulty in assessing whether neurological deterioration was due to the initial subarachnoid or intraprocedural hemorrhage.

Data collected from medical records included age; sex; medical history; family history of subarachnoid hemorrhage, UIA, or polycystic kidney disease; aneurysm size (dome and neck), location (internal carotid artery [ICA], anterior cerebral artery, middle cerebral artery, vertebrobasilar artery, or other), and presence of blebs; PHASES score (19); UCAS score (20); type of endovascular procedure (simple, double-catheter, balloon-assisted, or stent-assisted); cause of rupture (microcatheter, micro-guidewire, or coil); and type of guiding catheter (with or without balloon). Morbidity was defined as a decrease in the modified Rankin Scale score 30 days after treatment compared with the pre-treatment score.

Patients were divided into IPR and non-IPR groups on the basis of whether IPR occurred during coil embolization, as well as into BGC and non-BGC groups on the basis of whether guiding catheters with or without balloon were used.

### 2.2 Endovascular procedures

All endovascular procedures were performed under general anesthesia by or under the supervision of the attending physician. Patients received oral antiplatelet therapy (100 mg aspirin alone or 100 mg aspirin and 75 mg clopidogrel) before the procedure. Heparin was intravenously administered to maintain the activated clotting time at twice the normal value. Although the selection of the guiding catheter was based on the surgeon’s preference, BGCs (8 or 9F, Optimo, Tokai Medical Inc., Aichi, Japan, Merci Guiding system, FlowGate, Stryker, Kalamazoo, MI, United States) were the first-line guiding catheters for all anterior circulation aneurysms. In the case of tortuous vessel anatomy, as judged by the operator, even in the anterior circulation, or in the case of posterior circulation aneurysms, non-BGCs (6–8F guiding catheter or sheath) were used. We defined balloon-assisted coiling as coil embolization during which the balloon was inflated. We always prepared the microballoon on a tray regardless of the procedure performed (with or without balloon-assisted coiling).

### 2.3 Diagnosis and management of IPR

IPR was diagnosed by visualizing contrast extravasation. Immediately after IPR identification, the guiding catheter-mounted balloon was inflated to control bleeding. Blood pressure was decreased to normal, and protamine sulfate (30–40 mg) was administered. Additional coil placement was performed until the bleeding stopped, and cone-beam computed tomography was performed to evaluate the degree of subarachnoid hemorrhage and brain swelling.

### 2.4 Statistical analysis

Continuous variables are expressed as median values (interquartile range) and categorical variables as numbers (%). Statistical significance was assessed using the Wilcoxon rank-sum test for continuous variables and Fisher’s exact test for categorical variables. The association between baseline factors and IPR was analyzed using univariate and multivariate logistic regression analyses adjusted for confounding factors. Because of the extremely small number of IPR cases (*n* = 19) in this study, the adjustments were limited to age and sex. The association between aneurysm location and IPR was evaluated with ICA aneurysms without posterior communicating artery (PcomA) involvement as the reference. In the analysis of the association between adjunctive techniques and IPR, the simple technique was considered as the reference. In all analyses, a *p*-value <0.05 was considered statistically significant. All statistical analyses were performed using StataCorp 2019 (version 16.1; StataCorp LLC, College Station, TX, United States).

## 3 Results

### 3.1 Patient characteristics

**Figure 1:**
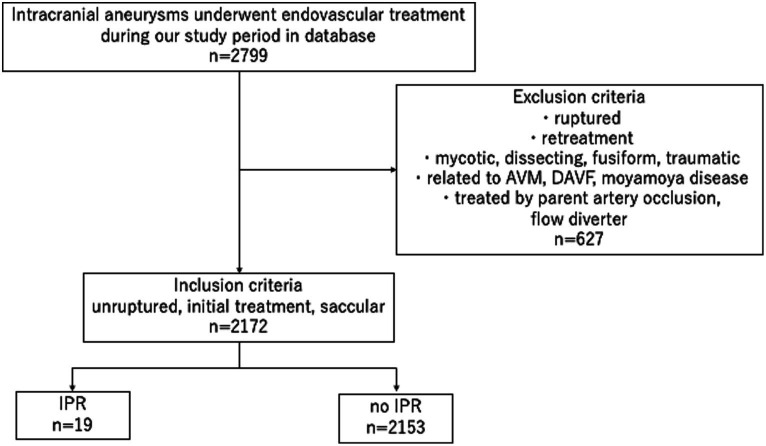
Study flowchart. AVM: arteriovenous malformation, DAVF: dural arteriovenous fistula, IPR: intraprocedural rupture.

Of the 2,799 aneurysms treated in our hospitals during the study period, 2,172 UIAs in 2026 patients were included in this study (Figure 1). Table 1 summarizes the patients’ demographic characteristics. There were no significant differences in medical or family histories between the IPR and non-IPR groups. BGCs were used for 1,082 aneurysms and non-BGCs for 1,090 aneurysms.

**Table 1:** Patient characteristics.

Characteristics	Total	IPR (−)	IPR (+)	*p*-value
Total no. of patients	2026	2007	19	
Age, y	61 (52–69)	61 (52–69)	64 (58–71)	0.29
Female sex	1,503 (74.2)	1,487 (74.1)	16 (84.2)	0.43
Medical history				
Hypertension	889 (43.9)	878 (43.7)	11 (57.8)	0.25
Diabetes mellitus	107 (5.3)	106 (5.3)	1 (5.2)	1.00
Dyslipidemia	386 (19.1)	384 (19.1)	2 (10.5)	0.56
Ischemic stroke	44 (2.2)	43 (2.1)	1 (5.2)	0.34
Cerebral hemorrhage	5 (0.2)	5 (0.2)	0	1.00
SAH	19 (0.9)	19 (0.9)	0	1.00
PKD	20 (1.0)	20 (1.0)	0	1.00
Smoking				0.51
None	1,358 (67.0)	1,347 (67.1)	11 (57.8)	
Current smoker	334 (16.5)	329 (16.4)	5 (26.3)	
Former smoker	334 (16.5)	331 (16.5)	3 (15.7)	
Alcohol consumption	265 (13.1)	262 (13.1)	3 (15.7)	0.73
Family history				
SAH	263 (13.0)	261 (13.0)	2 (10.5)	1.00
UIA	52 (2.6)	52 (2.6)	0	1.00
PKD	7 (0.3)	7 (0.3)	0	1.00

IPR: intraprocedural rupture, SAH: subarachnoid hemorrhage, PKD: polycystic kidney disease, UIA: unruptured intracranial aneurysm. Continuous variables are presented as medians (interquartile ranges), while categorical variables are presented as numbers (%). *p*-values were calculated using Wilcoxon’s rank-sum test for continuous variables and Fisher’s exact test for categorical variables.

The overall IPR rate was 0.8% (19/2172). The aneurysm morphological and surgical characteristics are shown in Table 2. The most common location of IPR was the anterior communicating artery (AcomA), followed by PcomA. The median size of the aneurysms with IPR was 4.7 mm (interquartile range, 3.6–6.5), with 10 aneurysms smaller than 5 mm, 7 sized 5–9 mm, and 2 larger than 10 mm. The double-catheter (761, 35.3%) and simple (662, 30.7%) techniques were most frequently used. There was no significant difference in the IPR rate between the non-BGC (*n* = 9) and BGC (*n* = 10) groups (0.8% vs. 0.9%, *p* = 0.822).

**Table 2:** Morphological and surgical characteristics.

Baseline factors	IPR (−)	IPR (+)	*p*-value
Total no. of aneurysms	2,153	19	
Bleb (yes)	489 (22.7)	9 (47.4)	0.023
Multiple aneurysms (yes)	651 (30.2)	5 (26.3)	0.810
Maximum size of aneurysm	5.7 (4.6–7.4)	4.7 (3.6–6.5)	0.052
Neck size	4.2 (3.4–5.3)	3.1 (2.8–4.6)	0.003
Location of aneurysm			0.008
ICA (without PcomA)	937 (43.5)	3 (15.8)	
PcomA	396 (18.4)	6 (31.6)	
AcomA	276 (12.8)	8 (42.1)	
MCA	235 (10.9)	1 (5.3)	
VABA	231 (10.7)	1 (5.3)	
Other	78 (3.6)	0	
PHASES score	7 (4–9)	8 (7–9)	0.18
UCAS score	4 (2–6)	5 (4–6)	0.028
Treatment technique			0.70
Simple	662 (30.7)	6 (31.6)	
Balloon-assisted	394 (18.3)	2 (10.5)	
Double-catheter	761 (35.3)	9 (47.4)	
Stent-assisted	336 (15.6)	2 (10.5)	

PcomA: posterior communicating artery, AcomA: anterior communicating artery, MCA: middle cerebral artery, VABA: vertebrobasilar artery; IPR: intraprocedural rupture; ICA, internal carotid artery. Continuous variables are presented as medians (interquartile ranges), while categorical variables are presented as numbers (%).

### 3.2 Risk factors for IPR

Univariate logistic regression analyses showed that the presence of blebs, small neck size, location in PcomA and AcomA, and UCAS prediction scores were associated with IPR (*p* < 0.05). In the multivariate logistic regression analysis adjusted for age and sex, the presence of blebs (odds ratio [OR]: 3.03, 95% confidence interval [CI]: 1.21–7.57, *p* = 0.017), small neck size (OR: 0.56, 95% CI: 0.37–0.85, *p* = 0.007), and location in the PcomA (OR: 4.92, 95% CI: 1.20–20.19, *p* = 0.027) and AcomA (OR: 12.09, 95% CI: 2.99–48.80, *p* < 0.001), compared with aneurysms in the ICA without PcomA involvement, were significantly associated with IPR (Table 3).

**Table 3:** Univariate and multivariate logistic regression analyses adjusted for age and sex.

Baseline factors	Univariate		Multivariate	
	OR (95% CI)	*p*-value	OR (95% CI)	*p*-value
Bleb (yes)	3.06 (1.23–7.57)	0.015	3.03 (1.21–7.57)	0.017
Multiple aneurysms (yes)	0.82 (0.29–2.29)	0.711	0.76 (0.27–2.14)	0.608
Maximum size of aneurysm	0.85 (0.68–1.06)	0.154	0.83 (0.66–1.05)	0.125
Neck size	0.57 (0.38–0.87)	0.010	0.56 (0.37–0.85)	0.007
Location of aneurysm				
ICA (without PcomA)	Reference	Reference	Reference	Reference
PcomA	4.73 (1.17–19.01)	0.028	4.92 (1.20–20.19)	0.027
AcomA	9.05 (2.38–34.35)	0.001	12.09 (2.99–48.80)	<0.001
MCA	1.33 (0.14–12.83)	0.806	1.50 (0.15–14.68)	0.728
VABA	1.35 (0.14–13.05)	0.794	1.68 (0.17–16.70)	0.656
Other	–	–	–	–
PHASES score	1.09 (0.95–1.25)	0.196	1.09 (0.94–1.26)	0.228
UCAS score	1.18 (1.01–1.39)	0.037	1.18 (0.99–1.41)	0.064
Treatment technique				
Simple	Reference	Reference	Reference	Reference
Balloon-assisted	0.56 (0.11–2.79)	0.479	0.55 (0.11–2.75)	0.467
Double-catheter	1.30 (0.46–3.69)	0.615	1.30 (0.46–3.67)	0.621
Stent-assisted	0.66 (0.13–3.27)	0.608	0.67 (0.13–3.34)	0.624

Continuous variables are presented as medians (interquartile ranges), while categorical variables are presented as numbers (%). OR: odds ratio, CI: confidence interval, PcomA: posterior communicating artery, AcomA: anterior communicating artery, VABA: vertebrobasilar artery, ICA: internal carotid artery, MCA: middle cerebral artery.

### 3.3 Timing and management of the IPR

As shown in Table 4, IPR occurred in 2 aneurysms during access and in 17 aneurysms during coiling. The IPR points were at the aneurysm neck (*n* = 1) and dome (*n* = 18). Thirteen ruptures were caused by the coil because of inappropriate microcatheter tip positioning or shape in the aneurysm sac, and five were caused by the microcatheter itself. One was caused by the microguidewire.

**Table 4:** Characteristics, management, and outcomes of IPR (*n* = 19).

Characteristics	*n* (%)
Timing of perforation	
Access	2 (10.5)
Coil replacement	
Framing	3 (15.7)
Filling	14 (73.6)
Cause of IPR	
Coil	13 (68.4)
Microguidewire	1 (5.2)
Microcatheter	5 (26.3)
Rupture point	
Neck	1 (5.2)
Dome	18 (94.7)
Additional therapy	
EVD	4 (21.0)
Clipping	1 (5.2)
Complication	
Symptomatic vasospasm	3 (15.7)
Hydrocephalus	3 (15.7)
Ischemia	
Temporary	2 (10.5)
Permanent	2 (10.5)
Clinical outcome	
Morbidity	3 (15.7)
Mortality	1 (5.2)

EVD: external ventricular drainage; IPR: intraprocedural rupture.

Additional surgical procedures were performed in five patients. One patient underwent hematoma evacuation, followed by clipping of the middle cerebral artery in the hybrid operating room. The patient was discharged 10 days after the procedure without any deficits. Four patients underwent ventriculostomy to control intracranial pressure. One patient died because of difficulty in controlling aneurysmal bleeding. In this case, a non-BGC was used, and an unsuccessful attempt was made to advance the microballoon across the neck of the aneurysm. In addition, because the contralateral A1 was large, balloon occlusion of the ipsilateral A1 did not result in hemostasis.

### 3.4 Clinical outcomes after IPR

Three patients (15.7%) had symptomatic vasospasms. Thromboembolic events occurred in four (21.0%) patients, two (10.5%) cases resulted in temporary neurological deficit, and two (10.5%) cases resulted in permanent disability. The overall morbidity and mortality rates after IPR were 15.7% (*n* = 3) and 5.2% (*n* = 1), respectively.

There was a significant difference in the morbidity– mortality rate after IPR between the non-BGC and BGC groups (44.4% vs. 0%, *p* = 0.033). None of the patients in the BGC group experienced any neurological deterioration or severe headaches. In contrast, the morbidity and mortality rates in the non-BGC group were 33% and 11%, respectively (Table 5).

**Table 5:** Outcomes related to balloon guide catheter use.

	BGC	Non-BGC	*p*-value
Total no. of aneurysms	1,082	1,090	
IPR	10 (0.9)	9 (0.8)	0.822
Clinical outcome of IPR			0.033
Good	10	5	
Morbidity/mortality	0	4	

BGC: balloon guide catheter, IPR: intraprocedural rupture. Morbidity was defined as a decrease in the modified Rankin scale score 30 days after treatment compared to the pre-treatment score. *p*-values were calculated using Fisher’s exact test. Statistical significance was set at a two-tailed *p* value < 0.05.

## 4 Discussion

In this study, we found that the presence of blebs, small neck size, and aneurysm location in PcomA and AcomA were independent predictors of IPR in patients with UIAs. Furthermore, the use of BGCs was associated with favorable outcomes after IPR.

Aneurysm rupture during endovascular treatment is a potentially serious event, with reported periprocedural mortality and morbidity rates as high as 63% (1–8, 21, 22). In previous studies, the reported IPR rate ranged from 1% to 10% (1–18). The incidence of rupture is higher in ruptured aneurysms than in UIAs (3, 4, 9–12), with a reported IPR rate of 2.6%–9.8% in patients with ruptured aneurysms (3–6, 9, 11–13, 21) and 0.5%–3.8% in those with UIAs (1–4, 7–9, 11, 12, 14–17). Ruptured intracranial aneurysms are deemed to have a higher risk for IPR than UIAs due to the vulnerability of the aneurysmal wall. Although the IPR rate in our study (0.8%) was comparable to or lower than that in previous studies of UIAs, the risk for IPR remains due to the overall fragility of aneurysms and the nature of the procedure.

### 4.1 Risk factors

In this study, AcomA and PcomA aneurysms were independent risk factors for IPR. Numerous previous studies have reported similar findings for AcomA aneurysms (4, 6, 8, 10, 12, 16, 17), including two recent studies specifically limited to UIAs in which AcomA aneurysms were identified as an independent risk factor in multivariate analyses (8, 16). Aneurysms in this region are distal and tortuous, making microcatheter navigation unstable and often highly technically challenging (23). These anatomical features may reflect the difficulty of the treatment. In contrast, only one study has reported that PcomA aneurysms are statistically at risk for IPR (18). In the present study, PcomA aneurysms had the second highest incidence of intraoperative rupture after AcomA aneurysms; however, the risk may have been overestimated because PcomA aneurysms were statistically analyzed in comparison with ICA aneurysms that did not involve PcomA. As shown in the ISUIA and UCAS trials, PcomA aneurysms have a high risk of spontaneous rupture, which may be related to the high rate of IPR (24).

Irregular aneurysm morphology, including blebs, is associated with aneurysm rupture. Studies on both ruptured and unruptured aneurysms have reported that blebs are a risk factor for IPR, and the results of our study confirmed this (6, 17, 25, 26). In the current study, a narrow aneurysm neck was also an independent risk factor for IPR. This is the first time a study of UIAs has found a narrow neck to be a risk factor. Although similar findings have been reported for ruptured cerebral aneurysms (13), other studies have reported a wide neck as a risk factor (5). Aneurysms with narrow necks restrict catheter movement and may result in direct pressure on the aneurysmal wall owing to the coil. Although small aneurysm size was not found to be a significant risk factor in this study, several prior studies have reported contrasting results, and the reason may be the same as that for narrow aneurysm neck (6, 8–10, 17).

### 4.2 Role of BGCs

Although no significant difference was observed in the incidence of IPR between the BGC and non-BGC groups, the use of BGCs was associated with good clinical outcomes, without recorded cases of morbidity or mortality. The microballoon was prepared on a tray in all cases with or without balloon-assisted coiling; however, it took longer to guide the microballoon to the aneurysm neck during IPR in cases without balloon-assisted coiling. Therefore, BCGs may have achieved an early hemostatic effect. In addition, no complications, such as vessel dissection or hemodynamic infarction, were observed with the use of BGCs.

**Figure 2:**
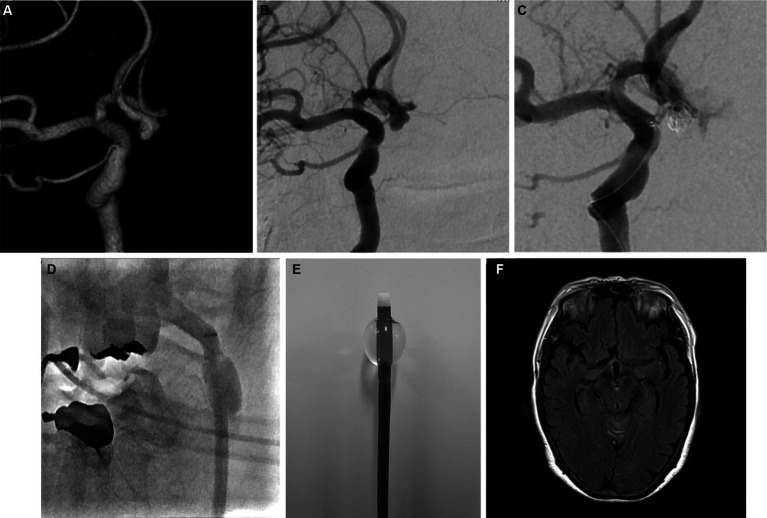
Representative pre- and postprocedural images. **(A)** Three-dimensional and **(B)** digital subtraction angiography (DSA) images showing an unruptured anterior communicating aneurysm. **(C)** DSA image showing active contrast extravasation due to aneurysm perforation. **(D)** Angiography image showing proximal flow control with balloon guiding catheter during intraprocedural rupture. **(E)** Inflated balloon guiding catheter (Flowgate2; Stryker, United States). **(F)** Magnetic resonance image (axial view) showing no subarachnoid bleeding in the basal cistern 1 day after perforation.

BGCs have been widely used following reports on their benefits in mechanical thrombectomy (27). The device is now considered the standard guiding system for thrombectomy in many centers to avoid trapped thrombus migration. Although the use of BGCs is widely accepted in neurointerventional procedures, this guiding system is not commonly used to treat cerebral aneurysms. We used an 8F BGC for flow control in cases of aneurysm IPR. The balloon can be inflated immediately, and flow control can be achieved within seconds. Using proximal flow control, additional coils can be advanced into the aneurysm until the bleeding site is completely occluded. The balloon is typically deflated after 2–3 coils are placed to assess angiographic hemostasis. In most cases, hemostasis was achieved within 5 min. Subarachnoid hemorrhage in this group was usually minimal, and follow-up computed tomography scans the following day showed blood washout in most cases (Figure 2).

Overall, the system is simple, does not require additional techniques, and allows for immediate control of bleeding. A disadvantage is that the system is not suitable for posterior circulation or tortuous ICA aneurysms because of the size of the existing balloon guiding systems.

### 4.3 Limitations

This study has several limitations. First, the retrospective study design may have introduced selection bias and affected the generalizability of our findings. Second, the number of patients with IPR was small, which may have limited the statistical power to detect significant differences between the groups. Furthermore, given the small number of IPR cases, the effect of several measured and unmeasured confounders was not assessed in this study. Third, this was an exploratory study to identify potential risk factors for IPR in UIAs. Further research is required to assess the validity of our findings. Finally, historical improvements in antiplatelet management, mechanical devices, and surgical techniques may have had a positive effect on the results of this study.

## 5 Conclusion

Aneurysms located in AcomA and PcomA, as well as aneurysms with blebs and small necks were identified as independent risk factors for IPR. Our results showed that BGCs may prevent serious clinical deterioration after IPR during coil embolization.

## Data availability statement

The raw data supporting the conclusions of this article will be made available by the authors, without undue reservation.

## Ethics statement

The studies involving humans were approved by the Ethics Committee at the Jikei University School of Medicine. The studies were conducted in accordance with the local legislation and institutional requirements. Written informed consent for participation in this study was provided by the participants’ legal guardians/next of kin.

## Author contributions

KA: Conceptualization, Data curation, Formal analysis, Investigation, Methodology, Project administration, Resources, Writing – original draft, Writing – review & editing. YM: Data curation, Investigation, Supervision, Writing – original draft, Writing – review & editing. YT: Investigation, Methodology, Writing – original draft, Writing – review & editing. TI: Data curation, Supervision, Writing – review & editing. KI: Data curation, Writing – review & editing. MF: Data curation, Writing – review & editing. NK: Data curation, Writing – review & editing. IK: Data curation, Writing – review & editing. KN: Data curation, Writing – review & editing. GN: Data curation, Writing – review & editing.
